# Microsatellite Typing of Clinical and Environmental *Cryptococcus neoformans* var. *grubii* Isolates from Cuba Shows Multiple Genetic Lineages

**DOI:** 10.1371/journal.pone.0009124

**Published:** 2010-02-09

**Authors:** Maria-Teresa Illnait-Zaragozi, Gerardo F. Martínez-Machín, Carlos M. Fernández-Andreu, Teun Boekhout, Jacques F. Meis, Corné H. W. Klaassen

**Affiliations:** 1 Instituto Pedro Kouri, Havana, Cuba; 2 Centraalbureau voor Schimmelcultures, Utrecht, The Netherlands; 3 Department of Medical Microbiology and Infectious Diseases, Canisius Wilhelmina Hospital, Nijmegen, The Netherlands; Pasteur Institute, France

## Abstract

**Background:**

Human cryptococcal infections have been associated with bird droppings as a likely source of infection. Studies toward the local and global epidemiology of *Cryptococcus* spp. have been hampered by the lack of rapid, discriminatory, and exchangeable molecular typing methods.

**Methodology/Principal Findings:**

We selected nine microsatellite markers for high-resolution fingerprinting from the genome of *C. neoformans* var. *grubii*. This panel of markers was applied to a collection of clinical (n = 122) and environmental (n = 68; from pigeon guano) *C. neoformans* var. *grubii* isolates from Cuba. All markers proved to be polymorphic. The average number of alleles per marker was 9 (range 5–51). A total of 104 genotypes could be distinguished. The discriminatory power of this panel of markers was 0.993. Multiple clusters of related genotypes could be discriminated that differed in only one or two microsatellite markers. These clusters were assigned as microsatellite complexes. The majority of environmental isolates (>70%) fell into 1 microsatellite complex containing only few clinical isolates (49 environmental versus 2 clinical). Clinical isolates were segregated over multiple microsatellite complexes.

**Conclusions/Significance:**

A large genotypic variation exists in *C. neoformans* var. *grubii*. The genotypic segregation between clinical and environmental isolates from pigeon guano suggests additional source(s) of human cryptococcal infections. The selected panel of microsatellite markers is an excellent tool to study the epidemiology of *C. neoformans* var. *grubii.*

## Introduction

Cryptococcosis ranks as one of the three common life-threatening opportunistic infections in persons with AIDS [1]. Global estimates indicate more than 900,000 annual cases, causing an estimated 624,700 deaths [2]. Other patient groups with impaired T-cell function have an up to 6% lifetime risk of developing clinically manifest cryptococcosis [3]. Seventy species belonging to the genus *Cryptococcus* have been described, but only members of the *C. neoformans* complex are mostly associated with human infections. This species complex has been considered to contain two pathogenic species: *C. neoformans* involving the varieties *neoformans* (serotype D) and *grubii* (serotype A), and *C. gattii* (serotypes B and C) [4]–[7]. Six monophyletic lineages have been identified that also may represent species [4], [5], [8] as well as some hybrids [9]–[12].

In the Caribbean the disease appears to be not very common with an estimated 7800 patients and 4300 casualties reported annually [2]. In Cuba the disease was first reported in the early 1950s [13]. Since then sporadic cases of cryptococcosis were associated with alcoholism, organ transplants and immunological disorders. Since the first cases of AIDS in Cuba in 1986, the number of patients infected by this fungus has increased over the years. The annual number of infected individuals ranged from 8 to 15 cases per year [14] while a study of 211 serial autopsies of patients with HIV/AIDS infection in Cuba over a period of 10 years, showed that systemic or central nervous system cryptococcosis was a serious and common disorder in 29% of cases [15]. Up to now all clinical isolates from patients in Cuba have been identified as *C. neoformans* var. g*rubii* [16].

Multiple molecular typing methods have been described to study the epidemiology of *C. neoformans* complex. The most commonly used approaches to date involve AFLP, PCR fingerprinting and or PCR-RFLP approaches as well as mating- and/or serotype-specific PCRs [17]–[23]. These techniques have proven useful to discriminate between the different sero- and mating types but have not been shown to be very useful for discrimination within specific *C. neoformans* complex members and varieties. Multi-locus sequence typing (MLST) has been applied to collections of *C. neoformans* and *C. gattii* from various origins [5], [24], [25] but this technique is laborious, has a long turn-around time and is associated with significant costs. Microsatellites are increasingly popular molecular typing targets since they provide cost-effective genotyping with fast turn-around times. On a theoretical basis, and, as has been shown for other fungi, molecular typing by using microsatellites is more discriminatory than by using MLST [26]. Like MLST data, microsatellite typing data is transportable and exchangeable [27]. Here we describe the use of a 9-marker microsatellite panel consisting of 3 dinucleotide repeat markers, 3 trinucleotide repeat markers and 3 tetranucleotide repeat markers. Each panel of 3 markers was amplified using a multiplex multicolor PCR approach. Amplified products were analyzed on a high resolution capillary electrophoresis platform allowing precise determination of repeat numbers in each marker. We applied this panel to a collection of clinical and environmental *C. neoformans* var. *grubii* isolates from Cuba. Part of this work was presented at the 46th Interscience Conference on Antimicrobial Agents and Chemotherapy (ICAAC), San Francisco, 2006, Abstr. M904.

## Materials and Methods

### Ethics Statement

This research was approved by the Institutional Scientific and Ethical Committee of the Instituto Pedro Kouri, Havana, Cuba. All data were analyzed anonymously.

### Isolates

**Figure 1:**
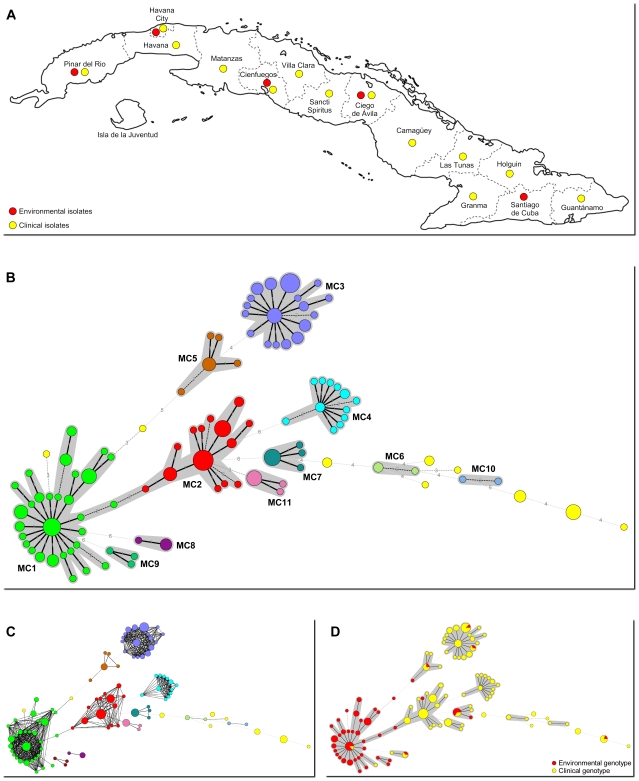
Origin of samples and relationships between genotypes. A: Map of Cuba. Colored dots indicate provinces from which clinical and/or environmental samples were available. B: Minimum spanning tree (MST) based on a multistate categorical analysis representing the genotypes of 190 *C. neoformans* var. *grubii* isolates from Cuba. Each circle represents a unique genotype. The size of the circle corresponds to the number of isolates with that genotype. Genotypes are linked to their closest relative. Numbers and connecting lines correspond to the number of different markers between genotypes. Genotypes with identical colors and connected by a shaded background are part of a microsatellite complex (MC). In yellow are unique genotypes that are not part of a MC. C: Same as B, but now showing cross-links between all genotypes that differ in no more than 2 markers. D: Same MST as in B, but now showing genotypes obtained from clinical and environmental samples.

A total of 190 clinical and environmental *Cryptococcus* isolates from the collection of the mycology laboratory at the Tropical Medicine Institute “Pedro Kourí”, were included in the study. Clinical strains (n = 122) were collected between 1987 and 2007, the large majority (91%) of isolates were from cerebrospinal fluid. The remaining isolates were from urine, blood, tissue biopsy and bronchoalveolar lavage samples. For ∼70% of all clinical isolates, information about the origin of the patients was available. Most patients (∼65%) were from Havana City. The remaining patients inhabited almost every province of Cuba (Figure 1). Approximately 77% of all patients were HIV positive. All clinical isolates were from different patients. Environmental isolates (n = 68) were isolated from pigeon guano collected in the period 1998 to 2007 from well distributed locations across Cuba (Figure 1). In case an environmental sample yielded multiple different colony morphologies suspected of being *Cryptococcus*, these were analyzed separately.

Species identification was initially performed by standard mycological methods [28] and confirmed with a commercial identification system (Auxacolor 2; Bio-Rad, Marnes-la-Coquette, France) as well as by using AFLP analysis.

### DNA Isolation

A suspension of freshly grown cells was prepared in lysis buffer (Roche Diagnostics, Almere, The Netherlands) and subjected to mechanical lysis in a MagNA Lyser for 30 s at 6500 rpm (Roche Diagnostics). Next, DNA was purified using a MagNAPure LC instrument in combination with a MagNAPure LC DNA Isolation Kit III as recommended (Roche Diagnostics). DNA yield and purity were estimated by UV absorbance measurements.

### Microsatellite Analysis

Candidate short tandem repeat markers were identified in the available genomic sequences from the H99 strain using the Tandem Repeats Finder software [29]. A 9 marker microsatellite panel consisting of 3 dinucleotide repeat markers, 3 trinucleotide repeat markers and 3 tetranucleotide repeat markers was selected from the candidate markers using previously described criteria [30]. PCR amplification primers for each of the markers are according to Table 1. Three subpanels (CNA2, CNA3 and CNA4 respectively) of 3 markers each were amplified using a multicolor multiplex PCR approach. Within each panel, one of the amplification primers carried a fluorescent label consisting of either FAM (6-carboxyfluorescein), HEX (hexachlorofluorescein) or TET (tetrachlorofluorescein). In addition to the amplification primers, each 50 µl amplification reaction contained approximately 1 ng of genomic DNA, 1 U FastStart Taq DNA polymerase (Roche diagnostics), 2 mM MgCl_2_ and 0.2 mM dNTP's in 1x reaction buffer (Roche diagnostics). The amplification profile consisted of a 10 min denaturation/activation step followed by 35 cycles of 94°C for 30 s, 60°C for 30 s and 72°C for 1 min. After an additional 10 min incubation at 72°C, the reactions were cooled to room temperature.

**Table 1:** Basic characteristics of the selected microsatellite markers.

Panel	Marker	Chr: position	Repeat unit	Labeled primer sequence (5′-3′)	Unlabeled primer sequence (5′-3′)*	Conc. (μM)	No. alleles (range)
CNA2	CNA2A	11: 389201–389350	CT	FAM- CGAGGTCATGTTGTGAGTCC	G TGACCGTCTCGTTCTTCTCA	0.3	15 (10–60)
	CNA2B	9: 191563–191711	TG	HEX- TCGTCAACGATGCAAGTCTC	GGGCCTGGGAAATAGGTAGA	0.3	6 (8–21)
	CNA2C	10: 307773–306912	TA	TET- AGAAGCACATGGGGAAAGG	GCGCAGTTTGAAGATGAGAA	1.0	16 (8–44)
CNA3	CNA3A	11: 1281429–12814716	CTA	FAM- ACCCCCTGCCCATCATA	GCACAGGCATAAAGCTAAGTGTGA	0.3	9 (19–69)
	CNA3B	4: 339525–339664	TCT	HEX- TGGGGATATCGATTCCTTCTC	GATTGGTATGGGAAGCGTTG	0.3	5 (5–17)
	CNA3C	7: 285123–285270	CCA	TET- TGGAAGAGGATGGAGCGTAT	GCATAGTTTATCGTTTTCTCTTTTC	0.3	10 (8–38)
CNA4	CNA4A	5: 233120–233445	TTAT	FAM- CGTCGAAGACTGCACAAAAA	GTTCTGTATGACAGGTCGCAAA	1.0	51 (15–119)
	CNA4B	4: 1021855–1022020	ATCC	HEX- CGGATGAGATGGAAAGAAGG	GTGCGTCTGTCAAAAGATTGC	0.3	10 (5–25)
	CNA4C	14: 131866–132031	TATT	TET- AGATGTCCTGGCGATGTTG	GAGGAGCAAGCAATCAAACC	0.3	11 (1–18)

* The underlined residue(s) are not a match to the genomic sequence. These were introduced to minimize the formation of minus A peaks, a well known PCR artifact that may complicate interpretation of the results [34].

### AFLP Analysis

Approximately 50 ng of genomic DNA was subjected to a combined restriction-ligation procedure containing 5 pmol of EcoR I adapter, 50 pmol Mse I adapter, 2 U of EcoR I (New England Biolabs, Beverly, MA, USA), 2 U of Mse I (New England Biolabs) and 1 U of T4 DNA ligase (Promega, Leiden, The Netherlands) in a total volume of 20 µl of 1x reaction buffer for 1 hour at 20°C. Next, the mixture was diluted five times with 10 mM Tris/HCl pH 8.3 buffer. Adapters were made by mixing equimolar amounts of complementary oligonucleotides (5′-CTCGTAGACTGCGTACC-3′ and 5′-AATTGGTACGCAGTC-3′ for EcoR I; 5′-GACGATGAGTCCTGAC-3′ and 5′-TAGTCAGGACTCAT–3′ for Mse I) and heating to 95°C, subsequently followed by cooling slowly to ambient temperature. One microliter of the diluted restriction-ligation mixture was used for amplification in a volume of 25 µl under the following conditions: 1 µM EcoR I primer with two selective residues (5′-Flu-GTAGACTGCGTACCCGTAC-3′), 1 µM MseI primer with one selective residues (5′-GATGAGTCCTGACTAAG-3′), 0.2 mM each dNTP and 1 U of Taq DNA polymerase (Roche Diagnostics) in 1x reaction buffer containing 1.5 mM MgCl_2_.

Amplification was done as follows. After an initial denaturation step for 4 min at 94°C in the first 20 cycles a touch down procedure was applied: 15 s denaturation at 94°C; 15 s annealing at 66°C with the temperature for each successive cycle lowered by 0.5°C and 1 min of extension at 72°C. Cycling was then continued for further 30 cycles with an annealing temperature of 56°C. After completion of the cycles an additional incubation at 72°C for 10 min was performed before the reactions were cooled to room temperature. The amplicons were then combined with the ET400-R size standard (GE Healthcare, Diegem, Belgium) and analyzed on a MegaBACE 500 automated DNA platform (GE Healthcare), according to the manufacturer's instructions.

### Capillary Electrophoresis

Following amplification, the reaction products were diluted 10-fold with distilled water. One µl of diluted products was combined with 0.25 µl of ET-ROX 550 size marker and 8.75 µl of distilled water. After a 1 min denaturation step at 94°C, the samples were quickly cooled to room temperature and injected onto a MegaBACE 500 automated DNA analysis platform equipped with a 48 capillary array as recommended by the manufacturer (GE Healthcare). Electropherograms were analyzed using Fragment Profiler 1.2 software (GE Healthcare). Assignment of repeat numbers was relative to the results obtained using the H99 strain, which was used as a control strain in all experiments. According to the genomic sequence, the genotype of the H99 strain was 27-20-20-57-17-14-55-19-16 for markers 2A-2B-2C-3A-3B-3C-4A-4B-4C respectively.

### Data Analysis

Typing data was imported into BioNumerics v5.0 software (Applied Maths, Sint-Martens-Latem, Belgium). Microsatellite data was analyzed using the multistate categorical similarity coefficient. Microsatellite complexes (MC's) were defined as groups of 2 or more genotypes differing by a maximum of 2 markers. AFLP data was analyzed by UPGMA clustering using the Pearson correlation coefficient.

## Results

A selection of 9 microsatellite markers was made from genomic sequences from *Cryptococcus neoformans* var. *grubii* strain H99. All markers proved to be polymorphic displaying a minimum of 5 and up to 51 different alleles per marker (Table 1). The specificity of the markers was tested by including *C. neoformans* var. *neoformans* isolates (serotype D) as well as *C. gattii* isolates (serotype B). None of these isolates yielded amplification products confirming the specificity of these primers for *C. neoformans* var. *grubii*.

In Table 2, the discriminatory power for each of the individual markers, panels and entire set of markers were calculated. When all markers are combined, the nine marker microsatellite panel yielded a discriminatory power of greater than 0.993. With this collection of 190 isolates, 104 different genotypes could be discriminated. The AFLP analysis confirmed that all isolates were *C. neoformans* var. *grubii* (not shown) in line with previous observations [16]. This panel of markers thus provides a highly discriminatory typing assay for *C. neoformans* var. *grubii*. The relationship between the different genotypes is illustrated in Figure 1. Within the large diversity of genotypes, complexes of closely related genotypes are recognized and indicated as microsatellite complexes (MC's). Within the MC's most of the genotypic variation is the result from variations in few microsatellite markers (Table 3). The most discriminatory marker was marker CNA4a. Elimination of this marker from the dataset resulted in a reduction of the number of different genotypes by approximately 50%, but did not affect the distribution of the isolates over the different MC's nor did it affect the segregation between the different MC's (results not shown). Eleven MC's are recognized containing up to 51 isolates each. Nine further genotypes (from 18 isolates) were observed that did not belong to a microsatellite complex, bringing the total number of different genogroups to 20. Four MC's (MC1-MC4) were the most prevalent and contain more than 70% of all isolates.

**Table 2:** Overview of the discriminatory power of the individual markers, panels of markers and the entire set of markers. Calculated values are based on the Simpson's index of diversity and are expressed in a value of ‘D’ [35].

Marker	D	Panel	D	Set	D
CNA2a	0.842	CNA2	0.906	CNA	0.993
CNA2b	0.789				
CNA2c	0.828				
CNA3a	0.282	CNA3	0.868		
CNA3b	0.618				
CNA3c	0.819				
CNA4a	0.972	CNA4	0.992		
CNA4b	0.712				
CNA4c	0.688				

**Table 3:** Signature profiles of the 4 most prevalent microsatellite complexes and the reference isolate H99 upon whose genome the selection of markers was made.

	CNA2a	CNA2b	CNA2c	CNA3a	CNA3b	CNA3c	CNA4a	CNA4b	CNA4c
MC1	53–60	11	9	0	13	35–38	85–108	8	5
MC2	11	8	22–25	0	14	16	30–37	8	5
MC3	10	12	10	0	14	14	66–78	6	9–11
MC4	12	10	8	0	5	8	103–119	5	1
H99	27	20	20	57	17	14	55	19	16

Not all markers yielded a PCR product with all of the isolates, especially markers CNA3a scored negative on a substantial part (85%) of the isolates. When a negative result was obtained, the particular marker was reamplified in a monoplex PCR reaction. When still negative, the marker was scored as “0”. Exclusion of marker CNA3a from the microsatellite panel did not influence the clustering of the isolates over the different MC's (not shown).

The distribution of the clinical and environmental isolates over the different MC's is shown in Table 4. Very interestingly, MC1 contained a large majority of isolates from environmental origin (>96% from pigeon guano) and only few human clinical isolates. This difference was highly significant (p<0,001). Though small in size, MC9 also exclusively contained isolates from environmental origin . The non environmental MC's were all found in HIV positive patients with one exception: MC6 contained 3 isolates and those were obtained from HIV negative patients.

**Table 4:** Distribution of isolates from clinical (including HIV status) or environmental origin over the 11 microsatellite complexes.

Complex	Total number of isolates	Env.	Clin.	HIV pos.	HIV neg.	HIV status unknown
MC1	51	49	2	2	0	0
MC2	32	2	30	16	6	8
MC3	39	2	37	27	9	1
MC4	14	0	14	14	0	0
MC5	8	2	6	5	0	1
MC6	3	0	3	0	3	0
MC7	9	5	4	4	0	0
MC8	4	1	3	3	0	0
MC9	3	3	0	0	0	0
MC10	2	0	2	1	1	0
MC11	7	0	7	4	1	2
other	18	4	14	9	5	0
Total	190	68	122	85	25	12

Env.: Environmental isolates; Clin.: Clinical isolates; pos.: positive; neg.: negative.

The temporal distribution of the largest MC's with clinical isolates showed their presence over prolonged periods of time since they were found repeatedly since the early years of the study and thus have been present for almost 2 decades.

## Discussion

A 9-marker microsatellite panel is described for high resolution sub typing of *C. neoformans* var. *grubii* isolates, one of the members of the *C. neoformans* complex, in particular serotype A isolates. This panel of markers provides a highly discriminatory panel allowing excellent discrimination between isolates from various origins. The numerical typing result allows easy storage into global databases as well as portability of the results.

From the results, it is obvious that certain genotypes appear to be more closely related to each other than to other genotypes. MC's were arbitrarily defined by genotypes differing in up to 2 microsatellite markers from each other. Within each MC, the amount of variation is attributable to only one or two microsatellite markers as the likely result of instability of these specific markers. This mostly involved the markers CNA2a, CNA2c and CNA4a. Not surprisingly, these are among the most discriminatory markers from the entire set (Table 2). Likewise, within an MC, there was very limited to no variation in the less discriminatory markers (Table 3). The difference between the MC's is attributable to multiple microsatellite markers (≥3 markers difference). Although MC1 and MC2 could be connected to each other by sequential genotypes differing in only 2 microsatellite markers, there appears to be no close relationship between these MC's (Figure 1C; Table 3) and these were therefore considered to reflect different unrelated MC's.

One marker that was selected from the H99 genome (CNA3a) did not yield an amplification product in 85% of all tested isolates. This could be the result of an actual deletion of this locus in the genome from these isolates or it could be the result of one or more sequence polymorphisms underneath either of the two amplification primers. Alternatively, the size of the amplified fragment could be beyond the reach of the size marker on the capillary electrophoresis runs. This was not further investigated. Whether or not this observation is specific for the Cuban population of *C. neoformans* var. *grubii*, which may very well be explained by the geopolitical isolation of this country, remains to be established.

In our study, the large majority of environmental isolates from pigeon droppings co-clustered in one MC (MC1) containing only very few isolates from human clinical samples. This MC was widespread in multiple environmental locations across Cuba. Vice versa, several of the other MC's predominantly contained isolates from human clinical samples and only few from environmental origin. Possibly, isolates from MC1 are more adapted to the pigeon host and may be less pathogenic for humans. Several MC's were identified that contained only clinical isolates and were never found in environmental samples which suggests the presence of additional niches of *C. neoformans* var. *grubii* that may cause human infections. One MC was identified (MC6) containing only isolates from humans without HIV infection. This may indicate that this particular genotype may be more virulent to humans but this clearly needs more confirmation. However, despite careful selection of multiple colonies from environmental samples, we cannot rule out the possibility that there could have been a sampling bias towards genotypes that are more abundantly present in pigeon guano.

In this study, we used AFLP analysis for a dual purpose. Firstly to confirm the identification of the isolates as *C. neoformans* var. *grubii*, and secondly to validate the typing result of the microsatellite analysis. The specific combination of restriction enzymes and selective residues used here was reported before [17]. Interestingly, we found only limited genetic variation in this collection of isolates. AFLP showed no segregation between the isolates in MC1 and the majority of the other isolates (results not shown). On the other hand, a relatively small number of clinical isolates (11%) did indeed segregate into a separate AFLP cluster. This subdivision is fully supported by the microsatellite data since this AFLP cluster is recognized as a separate MC (MC4, Figure 1), well separated from the other MC's. Since AFLP analysis amplifies fragments from multiple random locations in the genome, this may point to relatively few or small genomic differences between MC1 isolates and the majority of the other isolates versus substantial genomic differences between the majority of the isolates and those from MC4. Probably, the discriminatory power of the AFLP analysis can be increased by testing other combinations of restriction enzymes and/or selective residues but this was not attempted.

*C. neoformans* var. *grubii* has since long been associated with bird droppings: according to the Centers for Disease Control and Prevention, people with weakened immune systems should avoid areas contaminated with bird droppings and contact with birds [31]. However, this assumed link has received little attention using molecular typing studies. Our results suggest that certain clinical isolates may originate from additional ecological niche(s). This is supported by recent evidence from Litvintseva et al. that environmental isolates from pigeon excreta were less pathogenic in a mouse model than isolates from human clinical samples [32]. In this prior work, neither AFLP nor MLST proved useful to distinguish between these clinical and environmental isolates. Instead, a retrotransposon based Southern blotting procedure enabled them to distinguish between individual isolates with identical AFLP/MLST genotypes. However, analysis of serial cultures showed that the genotypes were not stable over time, limiting the usefulness of this approach. In addition, the authors did not report specific genotypes being associated with either clinical or environmental isolates. Our results confirm the superiority of this panel of microsatellite markers as molecular typing targets for *C. neoformans* var. *grubii* and simultaneously allow distinguishing between isolates from clinical and environmental origin. The lack of temporal and spatial (east to west Cuba is 1000 km) variability suggest a clonal relationship between the majority (>70%) of isolates from pigeon guano in Cuba. This too may be explained by the isolated location of the country. In contrast, most clinical cases were from Havana City and these involved multiple different MC's additionally suggesting the presence of alternative sources for human infections.

The use of microsatellite markers in typing studies with *C. neoformans* var. *grubii* was reported before. Hanafy et al. reported a selection of 15 microsatellite markers for *C. neoformans* var. *grubii* of which only 3 proved to be polymorphic [33]. The PCR products were analyzed by agarose gel electrophoresis. This technique suffers from insufficient resolution and does not allow use of the full potential of microsatellite markers as molecular typing targets.

In summary, we have developed a novel approach for high resolution molecular sub typing of *C. neoformans* var. *grubii*. This will help the study of the global epidemiology of this opportunistic pathogenic yeast. We also show that microsatellites are excellent multilevel genotyping targets allowing recognition of individual genotypes as well as clusters of related genotypes. The selected microsatellite markers are sufficiently stable for use in long-term longitudinal studies. Finally, our results point to additional sources other than bird droppings as origin of human infections.
